# A Comparative Evaluation of SIRS, NEWS2, SOFA, and the Novel SOFA-2 Score for Sepsis Classification Agreement and Outcome Prediction

**DOI:** 10.3390/diagnostics16111579

**Published:** 2026-05-23

**Authors:** Jing Qin, Yuan Yan, Chao Wang, Xingyu Tao, Ziyi Wu, Bin Liu, Bailin Niu

**Affiliations:** Department of Critical Care Medicine, Chongqing Key Laboratory of Emergency Medicine, Chongqing Emergency Medical Center (Chongqing University Central Hospital), School of Medicine, Chongqing University, Jiangkang Road, Yuzhong District, Chongqing 400016, China; xl450810@163.com (J.Q.);

**Keywords:** sepsis, SIRS, NEWS2, SOFA, SOFA-2

## Abstract

**Background:** Sepsis, defined as life-threatening organ dysfunction caused by a dysregulated host response to infection, remains a leading cause of death worldwide. Its diagnostic criteria have evolved from Sepsis-1 (SIRS) to Sepsis-3 (SOFA). The recent introduction of the SOFA-2 score, an update to the original SOFA, warrants validation in specific patient populations and against other established scoring systems. This study aims to compare the performance of SIRS, NEWS2, SOFA, and the newly proposed SOFA-2 in the classification agreement and prognostic performance for sepsis in a cohort of patients with suspected infection. **Methods:** This retrospective study enrolled adults with suspected infection admitted to a tertiary emergency center (January 2024–February 2025). SIRS, NEWS2, SOFA, and SOFA-2 scores were calculated from admission data. Patients were stratified using established thresholds (SIRS ≥ 2, NEWS2 ≥ 5, SOFA ≥ 2, SOFA-2 ≥ 2). Concordance with Sepsis-3 (SOFA ≥ 2) and prognostic accuracy for 28-day mortality were evaluated using AUROC analysis. Score distributions and organ dysfunction patterns were compared. **Results:** Of 562 screened patients, 516 were included. For sepsis classification agreement, SOFA-2 showed excellent agreement with SOFA (kappa = 0.923) and higher specificity than SIRS and SOFA. For 28-day mortality prediction, SOFA-2 showed the numerically highest AUC (0.863, 95% CI: 0.830–0.892), demonstrating slightly better discrimination than SOFA (AUC:0.854, 95% CI: 0.820–0.883). Pairwise DeLong tests indicated no significant differences between SOFA-2 and SOFA (*p* = 0.160). At optimal cutoffs, SOFA-2 demonstrated higher specificity (89.08% vs. SOFA 78.59% vs. NEWS2 76.87% vs. SIRS 41.33%), while SOFA showed higher sensitivity (81.63% vs. SOFA-2 73.47%). Increasing SOFA-2 scores strongly correlated with higher in-hospital mortality and longer ICU stay (both *p* < 0.001). SOFA-2 reclassified respiratory and cardiovascular dysfunction with higher thresholds and greater granularity than SOFA. **Conclusions:** Based on our dataset, SOFA-2 demonstrates high diagnostic alignment with Sepsis-3 classification and higher specificity for mortality prediction, with slightly better discrimination compared to SOFA, NEWS2, and SIRS. While its slightly lower sensitivity may limit early risk stratification in some patients, its strong prognostic performance supports its utility for risk stratification. Multicenter studies are warranted to determine its role in future sepsis definitions.

## 1. Introduction

Sepsis, defined as life-threatening organ dysfunction caused by a dysregulated host response to infection, remains a leading cause of death worldwide since the first consensus definitions were established in 1991 [1]. The global burden of sepsis is immense, with an estimated 48 million cases of sepsis occurring each year and approximately 11 million sepsis-related deaths [2]. Thus, improving efficient recognition, accurate identification, and timely intervention is essential to enhance patient outcomes and reduce the global burden of sepsis.

The diagnostic criteria for sepsis have evolved over the past three decades. Sepsis-1, introduced in 1991, defined sepsis as systemic inflammatory response syndrome (SIRS) secondary to infection [3]. In 2001, Sepsis-2 expanded the criteria by incorporating additional clinical and laboratory parameters to facilitate bedside diagnosis [4]. The current Sepsis-3 definition, established in 2016, replaced SIRS with the Sequential Organ Failure Assessment (SOFA) score, emphasizing organ dysfunction as the core feature of sepsis, which was intended to improve specificity and clinical relevance [1].

Since its introduction in 1996, the SOFA score has been widely used for nearly 30 years. However, advances in critical care practices and the emergence of data-driven artificial intelligence have created a need for an updated tool that better reflects contemporary care settings. In response, Ranzani et al. (2025) recently introduced and validated the SOFA-2 score through an international expert consensus and data-driven approach, demonstrating improved accuracy in reflecting organ dysfunction in contemporary ICU patients and enhanced predictive performance for clinical outcomes [5].

Despite these advances, critical questions remain. While SOFA-2 has been validated in mixed ICU populations and initially explored in sepsis cohorts [6], its performance relative to other established tools—particularly SIRS and NEWS2—has not been systematically compared. NEWS2, although primarily developed as an early warning score for clinical deterioration, has demonstrated prognostic utility in patients with suspected infection and has been incorporated into recent guidelines due to its association with adverse outcomes [2]. qSOFA was initially proposed as a rapid screening tool to identify patients with suspected infection at high risk of poor outcomes, such as death or prolonged ICU stay [1]. However, subsequent analyses have demonstrated its limited sensitivity for sepsis detection. As highlighted in the 2021 [7] and 2026 [2] Surviving Sepsis Campaign (SSC) guidelines, qSOFA is no longer recommended as a sole screening tool due to its low sensitivity. Instead, it is primarily used for identifying patients at risk of poor outcomes, rather than for definitive sepsis identification.

The present study aims to address these gaps by comparing the classification agreement and prognostic performance of SIRS, NEWS2, SOFA, and the novel SOFA-2 score in a cohort of patients with suspected infection. By analyzing over 500 cases, we seek to: (1) evaluate the classification consistency and prognostic value of SOFA-2 relative to established scoring systems; (2) examine the distribution of organ dysfunction scores between SOFA and SOFA-2; and (3) explore the potential of SOFA-2 as a refined framework for future sepsis definitions and clinical assessment.

## 2. Methods

### 2.1. Study Design and Population

This single-center retrospective observational study consecutively screened adult patients (≥18 years) with suspected infection admitted to the emergency ward of Chongqing Emergency Medical Center between January 2024 and February 2025. Suspected infection was defined in accordance with Sepsis-3 criteria, as outlined by the Third International Consensus Definitions Task Force [1]. It refers to the co-occurrence of antibiotic administration and microbiological culture sampling. Specifically, if antibiotics were given first, culture samples must be obtained within 24 h. If the culture was obtained first, antibiotics must be initiated within 72 h [1].

Of 562 initially screened patients, 46 were excluded based on pre-specified criteria, leaving 516 patients for final analysis. Exclusion criteria were as follows: (1) equivocal baseline SOFA scores precluding accurate assessment (*n* = 11), for patients with chronic conditions, baseline SOFA was estimated using clinical history and laboratory data from a recent stable period (within 1–3 months prior to admission); (2) incomplete medical records with missing key variables for score calculation (*n* = 23); (3) absence of required laboratory tests within 24 h of sepsis diagnosis (*n* = 8); (4) age < 18 years (*n* = 1); (5) pregnancy (*n* = 1); (6) transfer from another hospital with length of stay >24 h (*n* = 1); and (7) do-not-resuscitate orders at admission (*n* = 1).

### 2.2. Data Collection

Demographic information, clinical parameters, laboratory biomarkers, and outcome data were extracted from electronic medical records. All variables were collected based on the worst values within the first 24 h of admission, defined as the values reflecting the most severe physiological derangement. The following data were collected: (1) Demographics: Age, sex; (2) Vital signs: Heart rate, respiratory rate, systolic and diastolic blood pressure, mean arterial pressure, temperature, oxygen saturation; (3) Laboratory biomarkers: White blood cell (WBC) count, hemoglobin (Hb), neutrophil count (N#) and percentage (N%), platelet count (PLT), lymphocyte count (Lym), C-reactive protein (CRP), procalcitonin (PCT), interleukin-6 (IL-6), lactate (Lac), alanine aminotransferase (ALT), aspartate aminotransferase (AST), total bilirubin (TBil), direct bilirubin (DBil), albumin (Alb), serum creatinine (Cr), blood urea nitrogen (BUN), prothrombin time (PT), activated partial thromboplastin time (aPTT), international normalized ratio (INR), fibrinogen (Fig), D-dimer; (4) Clinical data: Oxygenation index (OI, PaO_2_/FiO_2_), primary infection site (lung, abdominal cavity, urinary tract, skin/soft tissue, others), comorbidities. (5) Outcome data: ICU stays, 28-day mortality, in-hospital mortality.

### 2.3. Scoring Systems

All scores were calculated based on the worst values within the first 24 h of admission, using established criteria:

(1) SIRS score: Calculated according to the 1991 consensus conference definitions [3]. One point was assigned for each of the following: (1) temperature > 38 °C or <36 °C; (2) heart rate > 90 beats/min; (3) respiratory rate > 20 breaths/min or PaCO_2_ < 32 mmHg; (4) WBC > 12,000/mm^3^, <4000/mm^3^, or >10% immature bands. Total score range: 0–4.

(2) NEWS2 score: Calculated from six physiological parameters (respiratory rate, oxygen saturation, systolic blood pressure, heart rate, level of consciousness or new confusion [ACVPU scale], and temperature), according to Royal College of Physicians guidelines [8]. Total score range: 0–20.

(3) SOFA score (SOFA-1): Calculated using the original 1996 criteria [9], assessing six organ systems (respiration, coagulation, liver, cardiovascular, central nervous system, renal). Each system scored 0–4, with a total score range of 0–24.

(4) SOFA-2 score: Calculated according to the updated criteria published by Ranzani et al. (2025) [5]. This version incorporates contemporary treatment modalities (e.g., vasopressor type and dose, high-flow nasal oxygen) and alternative variables when primary measures are unavailable, with each of six organ systems scored 0–4 (total range 0–24).

For comparative analyses, patients were stratified using established diagnostic thresholds: SIRS ≥ 2, NEWS2 ≥ 5, SOFA-1 ≥ 2, and SOFA-2 ≥ 2. The ≥2 cutoff for SOFA-2 was adopted to maintain consistency with the Sepsis-3 definition based on SOFA ≥ 2, enabling parallel comparison between scoring systems.

### 2.4. Outcomes

The primary outcomes were as follows: (1) agreement between the four scoring systems and the Sepsis-3 definition (SOFA-1 ≥ 2 with suspected infection) for identifying sepsis; and (2) prognostic accuracy for predicting 28-day mortality. Secondary outcomes included in-hospital mortality, ICU stays, and the distribution of organ-specific dysfunction scores between SOFA-1 and SOFA-2.

### 2.5. Statistical Analysis

Statistical analyses were performed using SPSS (version 29.0, IBM Corp., Armonk, NY, USA), MedCalc (v20.0.3.0), GraphPad Prism (8.0.2), and R (v4.4.3, R Foundation for Statistical Computing, Vienna, Austria). Normality of continuous data was assessed using the D’Agostino-Pearson test. As most data were non-normally distributed, continuous variables were presented as medians with interquartile ranges (IQR) and compared using the Mann–Whitney U test (two groups) or Kruskal–Wallis test (multiple groups). Categorical variables were presented as counts (*n*) and percentages (%) and compared using the Chi-square test or Fisher’s exact test, as appropriate. Agreement between SOFA-1 and SOFA-2 for sepsis diagnosis (using the ≥2 threshold) was evaluated using Cohen’s kappa coefficient, with values interpreted as: <0.20 poor, 0.21–0.40 fair, 0.41–0.60 moderate, 0.61–0.80 good, and 0.81–1.00 excellent agreement.

The predictive performance of the four scoring systems for 28-day mortality was assessed by receiver operating characteristic (ROC) curve analysis. The area under the ROC curve (AUC) was calculated with 95% confidence intervals (CI). AUC values were interpreted as poor (0.6 to <0.7), adequate (0.7 to <0.8), good (0.8 to <0.9), and excellent (≥0.9). Optimal cut-off values were determined by maximizing the Youden index (sensitivity + specificity − 1). Sensitivity, specificity, positive predictive value (PPV), and negative predictive value (NPV) were reported at these cut-offs. DeLong’s pairwise tests were used for comparisons of AUCs between the scoring systems, and confidence intervals for sensitivity and specificity were also calculated. Restricted cubic spline (RCS) regression with three knots was used to explore potential non-linear relationships between SOFA-2 scores and ICU stays. A two-tailed *p*-value < 0.05 was considered statistically significant for all analyses. Patients with missing key variables required for SOFA, SIRS, or NEWS2 scores, or for primary outcomes, were excluded. Missing data for non-critical variables, such as laboratory markers not affecting the calculation of SOFA, SIRS, or NEWS2 scores, were handled using multiple imputation based on observed values.

## 3. Results

### 3.1. Clinical Characteristics of the Study Population

Patients were classified into sepsis and non-sepsis groups according to both SOFA-1 (≥2 points) and SOFA-2 (≥2 points) criteria. The baseline characteristics of the study population, stratified by both scoring systems, are presented in Table 1. Regarding demographic characteristics, both SOFA-1 and SOFA-2 criteria revealed significant differences between sepsis and non-sepsis patients in age, sex, and mortality rates (all *p* < 0.001). For clinical biomarkers, a notable discrepancy emerged between the two scoring systems. Under SOFA-2 classification, WBC count was significantly higher in septic patients compared to non-septic patients [11.90 (8.21, 17.10) vs. 10.70 (8.00, 14.11) × 10^9^/L, *p* = 0.01]. However, this difference was not statistically significant under SOFA-1 classification [11.61 (7.8, 16.85) vs. 10.97 (8.3, 14.33) × 10^9^/L, *p* = 0.173]. ALT showed no significant difference between groups under either scoring system (*p* > 0.05). Except for WBC and ALT, all other commonly used sepsis biomarkers demonstrated significant differences between septic and non-septic patients under both SOFA-1 and SOFA-2 criteria (all *p* ≤ 0.005). These included: Hb, N%, N#, PLT, Lym, CRP, PCT, IL-6, OI, Lac, AST, TBil, DBil, Alb, Cr, BUN, PT, aPTT, INR, fibrinogen, and D-dimer. Baseline characteristics of patients stratified by SIRS criteria are presented in Supplementary Table S1.

### 3.2. Distribution of Organ Dysfunction Scores Under SOFA-1 and SOFA-2 Criteria

The distribution of organ-specific dysfunction scores across all six organ systems, as assessed by both SOFA-1 and SOFA-2 criteria, is presented in Table 2. Distinct variations emerged in the scoring patterns for the respiratory and cardiovascular systems between the two versions. For respiratory dysfunction, SOFA-2 employs a higher threshold than the original SOFA-1. While the total number of patients with scores in the 0–1 range was similar between the two systems, the distribution within this range differed substantially. Specifically, the number of patients assigned a score of 1 was markedly lower under SOFA-2 compared to SOFA-1 (93 vs. 153 cases), with a corresponding increase in patients assigned a score of 0 (336 vs. 221 cases). This redistribution underscores the impact of SOFA-2’s refined thresholds for mild-to-moderate respiratory dysfunction.

Conversely, for cardiovascular dysfunction, SOFA-2 provides a more granular classification, particularly in the moderate-to-severe range. Although the total number of patients with scores in the 2–4 range was similar between the two systems, the distribution shifted significantly toward lower scores within this range. The number of patients assigned a score of 2 was substantially higher under SOFA-2 compared to SOFA-1 (86 vs. 17 cases), with corresponding reductions in patients assigned scores of 3 (27 vs. 52) and 4 (38 vs. 84). This reclassification reflects the incorporation of contemporary vasopressor practices into the SOFA-2 scoring algorithm. For the remaining organ systems (brain, liver, kidney, and hemostasis), the score distributions were broadly similar between SOFA-1 and SOFA-2, with only minor variations in case allocation across score categories.

### 3.3. Classification Agreement Among SIRS, SOFA-1, and SOFA-2 Criteria

**Figure 1:**
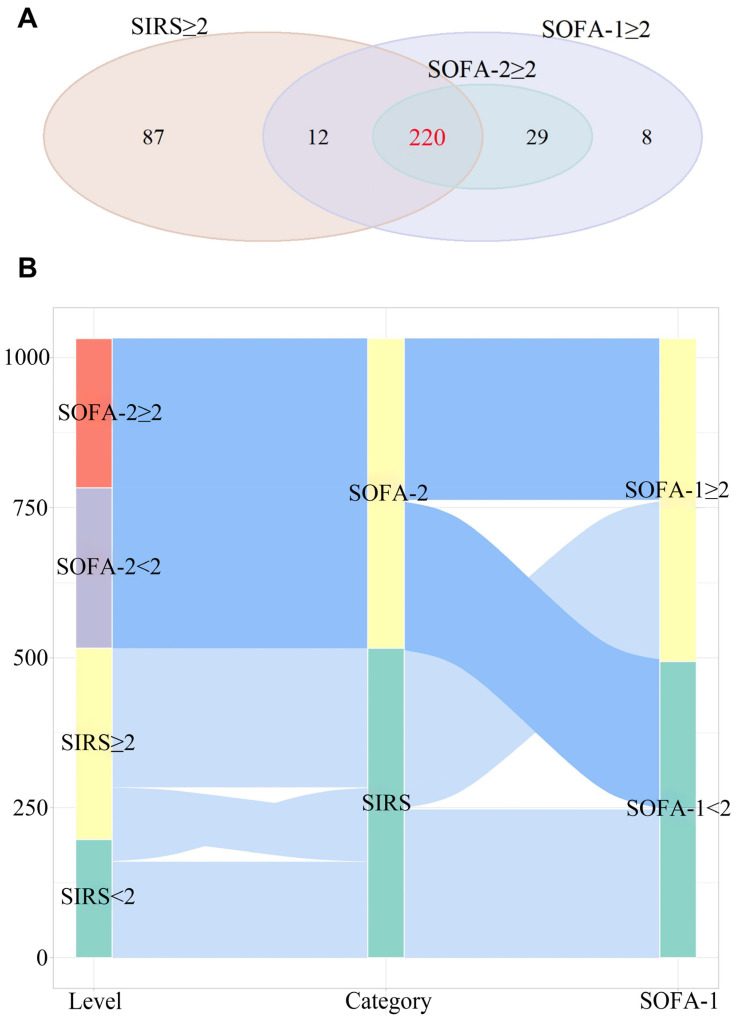
Classification of patients with suspected infection by SIRS, SOFA-1, and SOFA-2 criteria. Visualization includes a Venn diagram illustrating diagnostic overlaps, where the red number indicates the subset of patients achieving agreement across all three scoring systems (**A**), and a Sankey diagram depicting patient flow across the three scores (**B**).

Application of the established diagnostic thresholds yielded progressively smaller sepsis cohorts: 319 cases via SIRS, 269 via SOFA-1, and 249 via SOFA-2. This trend reflects the increasingly stringent specificity of the scoring criteria as they evolved from Sepsis-1 to the proposed SOFA-2 definition. The overlap in classification among the three scoring systems is illustrated in Figure 1 and Supplementary Figure S1. Using SOFA-1 as the reference standard, 269 patients were identified as septic, of whom 232 (86.2%) also met SIRS criteria, while 37 (13.8%) were SIRS-negative. Notably, all 249 patients categorized as septic by SOFA-2 (≥2 points) were also captured by SOFA-1, demonstrating complete concordance at this threshold. In contrast, the overlap between SOFA-1 and SIRS was only partial. To quantitatively assess the classification alignment, a Kappa consistency test was performed (Supplementary Table S2). The agreement between SOFA-2 and SOFA-1 was excellent, with a Cohen’s kappa of 0.923 (95% CI, 0.889–0.956; *p* < 0.001), indicating that the categorization of patients using SOFA-2 is highly consistent with the current Sepsis-3 standard. Supplementary Figure S1 provides a visual depiction of the patient flow and classification shifts among the four scoring systems using a Sankey diagram.

### 3.4. Prognostic Performance of the Four Scoring Systems for 28-Day Mortality

**Figure 2:**
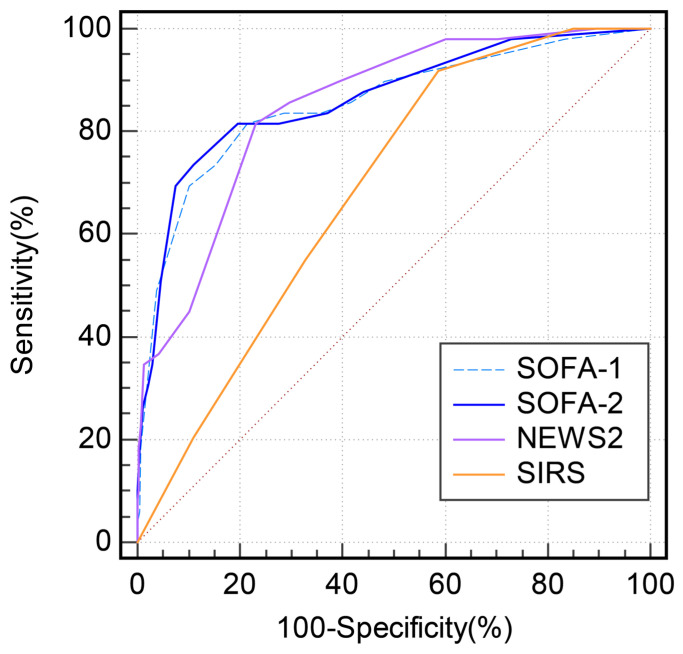
Receiver operating characteristic (ROC) curves of the four scoring systems (SOFA-1, SOFA-2, NEWS2, and SIRS) for predicting 28-day mortality in patients with suspected infection. SOFA-1 achieved an area under the curve (AUC) of 0.854 (95% CI: 0.820–0.883) with a sensitivity of 81.63%, specificity of 78.59% at a cutoff of 5; SOFA-2 achieved an AUC of 0.863 (95% CI: 0.830–0.892) with a sensitivity of 73.47%, specificity of 89.08% at a cutoff of 5; NEWS2 achieved an AUC of 0.849 (95% CI: 0.815–0.879) with a sensitivity of 81.63%, specificity of 76.87% at a cutoff of 7; and SIRS achieved an AUC of 0.686 (95% CI: 0.645–0.726) with a sensitivity of 91.84%, specificity of 41.33% at a cutoff of 1. All *p* < 0.001. Pairwise DeLong comparisons showed no significant differences between SOFA-1 and SOFA-2 (*p* = 0.160), SOFA-1 and NEWS2 (*p* = 0.850), or SOFA-2 and NEWS2 (*p* = 0.559), whereas comparisons involving SIRS were all significant (*p* < 0.001 for SOFA-1 vs. SIRS, SOFA-2 vs. SIRS, and NEWS2 vs. SIRS). The red dashed line represents the reference line with an area under the curve (AUC) of 0.5.

The predictive performance of SOFA-1, SOFA-2, NEWS2, and SIRS for 28-day mortality in patients with suspected infection was evaluated using ROC curve analysis (Figure 2). Among the four systems evaluated, SOFA-2 achieved the highest predictive accuracy (AUC: 0.863, 95% CI: 0.830–0.892; *p* < 0.001). At its optimal 5-point cutoff, SOFA-2 was distinguished by a superior specificity of 89.08% (95% CI: 85.9–91.8%), outperforming all other scores despite a moderate sensitivity of 73.47% (95% CI: 58.9–85.1%). SOFA-1 demonstrated comparable prognostic performance (AUC: 0.854, 95% CI: 0.820–0.883; *p* < 0.001), ranking second only to SOFA-2. Notably, at the same 5-point threshold, SOFA-1 retained a higher sensitivity (81.63%, 95% CI: 68.0–91.2%) than SOFA-2 but at the cost of lower specificity (78.59%, 95% CI: 74.6–82.2%). Similarly, NEWS2 exhibited a robust predictive capacity (AUC: 0.849, 95% CI: 0.815–0.879; *p* < 0.001), achieving an optimal balance at 7 points (sensitivity: 81.63%, 95% CI: 68.0–91.2%; specificity: 76.87%, 95% CI: 72.8–80.6%). In contrast, SIRS showed the weakest overall discrimination (AUC: 0.686, 95% CI: 0.645–0.726; *p* < 0.001), characterized by a high-sensitivity (91.84%, 95% CI: 80.4–97.7%) but low-specificity (41.33%, 95% CI: 36.8–45.9%) profile.

SOFA-2 demonstrated the highest specificity for mortality prediction (PPV:87.06%, NPV:77.05%), while SOFA-1 had a higher sensitivity (PPV:79.22%, NPV:81.05%). NEWS2’s performance was comparable (PPV:77.92%, NPV:80.71%). In contrast, SIRS, although highly sensitive, had the lowest PPV (61.02%) but high NPV (83.51%).

### 3.5. Distribution of Survival Outcomes Among Septic Patients Under Four Scoring Systems

**Figure 3:**
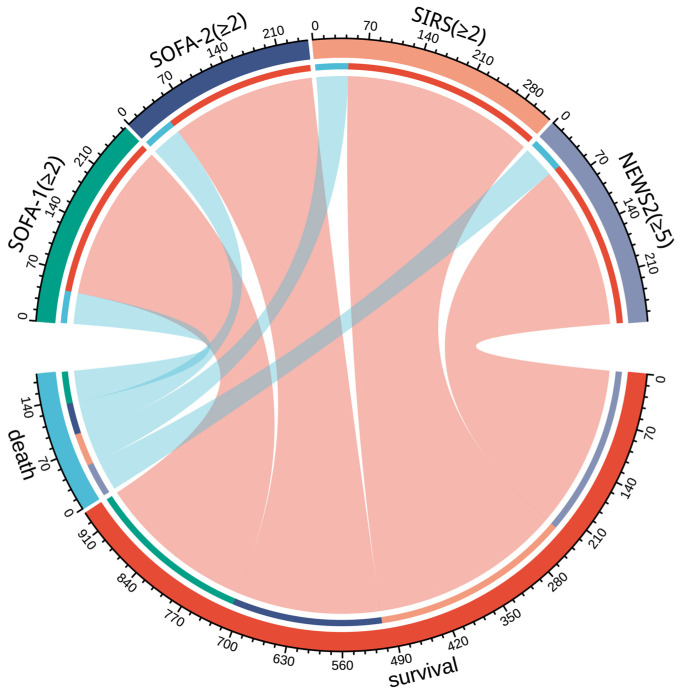
Distribution of 28-day survival outcomes among septic patients by four scoring systems. The chord diagram illustrates the flow of survivor and non-survivor status across septic patients meeting the diagnostic thresholds of SIRS (≥2), SOFA-1 (≥2), SOFA-2 (≥2), and NEWS2 (≥5). Using SIRS criteria, 319 patients were classified as septic, with 45 deaths. Under Sepsis-3 criteria (SOFA-1 ≥ 2), 269 patients were identified as septic, with 44 deaths. Applying SOFA-2 (≥2), 249 patients met the criteria for sepsis, with 43 non-survivors. Using NEWS2 (≥5), 278 patients were classified as high-risk, with 46 deaths. Each ribbon’s width is proportional to the number of patients, with blue segments representing non-survivors and red segments representing survivors at 28 days.

A chord diagram is presented in Figure 3 to illustrate the distribution of 28-day survival outcomes among patients classified as septic by each of the four scoring systems at their respective diagnostic thresholds. According to the SIRS criteria (≥2 points), 319 patients were classified as septic, with 45 deaths within 28 days. Under Sepsis-3 criteria (SOFA-1 ≥ 2), 269 patients were identified as septic, with 44 deaths. With the application of the proposed SOFA-2 threshold (≥2 points), 249 patients met the criteria for sepsis, including 43 non-survivors. For NEWS2 (≥5 points), 278 patients were classified as high-risk, with 46 deaths. At the recommended cutoffs, the sensitivity and specificity for each scoring system in our study cohort were as follows: SOFA-1 (≥2) showed a sensitivity of 85.71% and specificity of 58.24%, SOFA-2 (≥2) had a sensitivity of 83.67% and specificity of 62.96%, NEWS2 (≥5) showed the highest sensitivity at 89.80% with a specificity of 60.60%, and SIRS (≥2) had the highest sensitivity of 91.84% but a lower specificity of 41.33% (Figure 2). These values highlight the balance between sensitivity and specificity for each scoring system in predicting mortality. The chord diagram illustrates how survival outcomes overlap across the four systems, showing the degree of agreement in patient classification and mortality prediction.

### 3.6. Association Between SOFA-2 Score and In-Hospital Mortality

**Figure 4:**
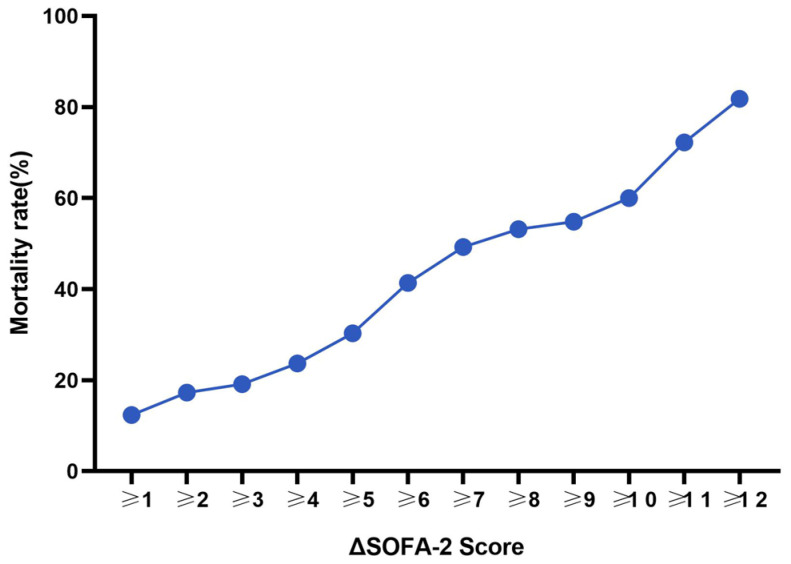
Association between SOFA-2 score and in-hospital mortality in patients with suspected infection. The line chart demonstrates a stepwise increase in mortality with ascending SOFA-2 score thresholds (from ≥1 to ≥12). Mortality rates rise progressively from 12.34% (score ≥ 1) to 81.82% (score ≥ 12), with a notable inflection point between thresholds ≥ 4 (23.67%) and ≥5 (30.30%). This dose–response relationship supports that higher SOFA-2 scores are associated with increased risk of in-hospital death.

In-hospital mortality exhibited a clear dose–response relationship with increasing SOFA-2 scores. As illustrated in Figure 4, mortality rates rose from 12.34% among patients with SOFA-2 ≥ 1 to 81.82% among those with SOFA-2 ≥ 12. The progression across intermediate thresholds was as follows: 17.27% (≥2), 19.16% (≥3), 23.67% (≥4), 30.30% (≥5), 41.38% (≥6), and 49.38% (≥7). A notable inflection point was observed between thresholds ≥4 and ≥5, where mortality increased by more than 10 percentage points (23.67% to 30.30%), suggesting a critical transition in risk stratification. Detailed mortality data for all thresholds are provided in Supplementary Figure S2. This progressive relationship suggests that SOFA-2 effectively captures the severity of organ dysfunction and its associated mortality risk.

### 3.7. Association of SOFA-2 Scores with ICU and Hospital Stays

**Figure 5:**
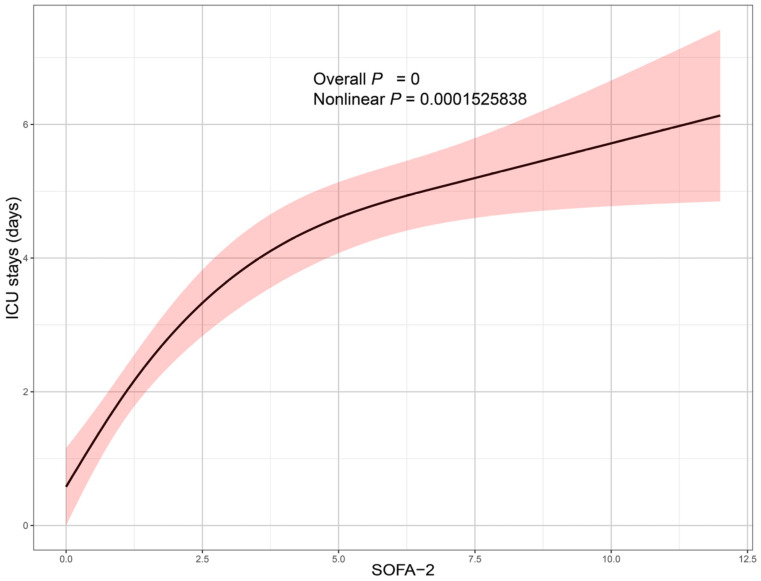
Restricted cubic spline analysis of the association between SOFA-2 scores and ICU stays. The line represents the fitted spline curve, with the pink shaded area indicating the 95% confidence interval. The analysis demonstrates a significant positive association between increasing SOFA-2 scores and longer ICU stay, with both overall (*p* < 0.001) and nonlinear (*p* < 0.001) effects reaching statistical significance.

Restricted cubic spline (RCS) analysis revealed a significant positive correlation between SOFA-2 score and ICU stays (Figure 5). The overall association was highly significant (*p* < 0.001), with a nonlinear component also reaching statistical significance (*p* < 0.001), indicating that the relationship is not simply linear. As SOFA-2 scores increased, ICU stays progressively extended, supporting the utility of SOFA-2 in reflecting disease severity. In addition, the correlation between SOFA-2 scores and total hospital length of stay was evaluated using a linear scatter plot analysis. As shown in Supplementary Figure S3, a weak but statistically significant positive correlation was observed between SOFA-2 scores and total hospital length of stay. This finding supports the overall trend observed in the ICU analysis, although the hospital-wide correlation did not exhibit the same nonlinear pattern identified by the RCS model.

## 4. Discussion

Delays in sepsis diagnosis are associated with a 7–10% increase in mortality risk per hour [10], underscoring the critical importance of early and accurate identification. The evolution of sepsis diagnostic criteria from Sepsis-1 to Sepsis-3 reflects this imperative, progressively shifting from the sensitive but non-specific SIRS criteria to the more organ-dysfunction-focused SOFA score [1]. Our results align with this paradigm shift, underscoring a transition from inflammation-centric criteria toward a more rigorous focus on infection-induced organ dysfunction. This transition enhances the clinical specificity of sepsis identification by focusing on organ dysfunction attributable to infection, rather than systemic inflammatory responses alone. Consistent with this framework, SIRS demonstrated the highest sensitivity (91.84%) but the lowest specificity (41.33%) for predicting 28-day mortality in patients with suspected infection, reflecting systemic inflammatory responses, with limited ability to specifically capture infection-associated organ dysfunction [11,12].

Sepsis-2 expanded the 1991 definition to include a broader list of clinical and laboratory indicators, introducing the SOFA score as a tool to quantify organ dysfunction [4]. However, SIRS remained central, diagnostic items became more complicated, and improvements in clinical utility were limited [13]. Therefore, our study focused on comparing SIRS, NEWS2, SOFA, and SOFA-2 rather than Sepsis-2. NEWS2, an internationally recommended early warning score developed by the Royal College of Physicians [8], demonstrated prognostic performance comparable to SOFA in our cohort, with an AUC of 0.849 for 28-day mortality prediction. This aligns with previous studies showing that NEWS2 is significantly associated with sepsis diagnosis within 24 h, ICU admission, and in-hospital mortality, with scores ≥ 5 indicating markedly increased risk of subsequent SOFA score elevation [14,15]. Although NEWS2 lacks specificity for sepsis diagnosis, it served as a prognostic benchmark for early warning and risk stratification in this study, rather than a primary diagnostic framework. While it shows strong prognostic value, particularly in emergency settings, it is more suited for early screening rather than defining sepsis. In contrast, SOFA and SOFA-2 are focused on quantifying organ dysfunction, making them more suitable for characterizing the core features of the sepsis definition.

The primary focus of this study was to evaluate the performance of the recently proposed SOFA-2 score against existing tools and to explore its potential as a candidate framework for future refinement of sepsis assessment. Our analysis elucidates key functional differences between the two versions. First, regarding prognostic performance, SOFA-2 showed excellent agreement with SOFA at the ≥2 threshold (kappa = 0.923), consistent with the recent validation by Wu et al. [6] who reported 96.8% concordance between the two versions, with only 2.2% of patients showing SOFA ≥ 2 but SOFA-2 < 2. Notably, all patients meeting the SOFA-2 threshold (≥2) were also identified by SOFA-1 (≥2), whereas a subset of patients classified as positive by SOFA-1 did not meet the SOFA-2 threshold. This finding suggests that applying SOFA-2 may reclassify some current Sepsis-3 cases below the diagnostic threshold and therefore warrants cautious interpretation. However, the small subset of patients who are SOFA-positive but SOFA-2-negative warrants further investigation, as these individuals may represent a group whose organ dysfunction, as defined by older parameters, is not captured by the contemporary, outcome-calibrated SOFA-2 thresholds.

Second, in prognostic assessment, at their respective optimal cut-off values, SOFA-2 was distinguished by its superior specificity (89.08%), markedly outperforming SOFA-1 (78.59%), NEWS2 (76.87%), and particularly SIRS (41.33%) in predicting 28-day mortality, while showing only a numerically higher AUC than SOFA. This pattern may reflect the recalibration of SOFA-2 toward contemporary outcome-based assessment, although the difference in discrimination between SOFA-2 and SOFA was not statistically significant. Conversely, SOFA exhibited higher sensitivity (81.63% vs. 73.47%), suggesting it may be better suited for initial screening, while SOFA-2 excels in risk stratification and prognostication. An important methodological consideration is the potential circularity arising from the use of SOFA-based Sepsis-3 criteria as the reference standard when comparing SOFA and SOFA-2. As SOFA-2 is conceptually derived from the original SOFA framework, this comparison may introduce incorporation bias. Therefore, the findings should be interpreted as an assessment of agreement and relative performance within a shared conceptual framework, rather than an independent validation.

Third, SOFA-2 demonstrated reclassification in both respiratory and cardiovascular dysfunction with higher thresholds and greater granularity compared to SOFA. This recalibration is particularly notable for the more precise classification of moderate-to-severe respiratory and cardiovascular dysfunction, which reflects current clinical practices. These recalibrations may better reflect contemporary respiratory support strategies (e.g., HFNO) and vasopressor practices, potentially improving clinical interpretability. Such recalibration may facilitate a more clinically relevant classification of organ dysfunction severity in contemporary practice, particularly in settings where respiratory support modalities and vasopressor strategies have evolved substantially since the original SOFA was introduced.

The strong dose–response relationships observed between SOFA-2 scores and both in-hospital mortality (Figure 4) and ICU stays (Figure 5) further support its association with disease severity. The nonlinear association with ICU stays, confirmed by restricted cubic spline analysis, highlights the complex relationship between organ dysfunction and healthcare resource utilization. However, ICU stays may also be influenced by competing clinical events and care processes, including early death, successful treatment, discharge strategies, and patient transfer decisions.

The expanding landscape of sepsis biomarkers highlights both opportunities and limitations. Classical markers such as procalcitonin, C-reactive protein, and lactate provide useful information but remain insufficient as standalone diagnostic indicators [16,17,18]. Consistent with recent guidelines [2], sepsis is a clinical syndrome that cannot be reliably defined using a single biomarker, underscoring the limitations of isolated indicators in capturing its biological complexity. In this context, multi-parameter approaches, which integrate dysfunction across multiple organ systems, may better reflect the pathophysiological basis of sepsis by addressing common underlying features of organ dysfunction. The SOFA score itself represents such a framework, integrating dysfunction across various organ systems. This multi-organ approach is essential for capturing the complexity of sepsis. Consistent with this concept, our previous work also demonstrated that the LIP score [19], which integrates lymphocyte count, INR, and procalcitonin, represents a promising screening tool for sepsis.

Beyond diagnosis, sepsis is increasingly recognized as a heterogeneous syndrome with diverse immunological and biological profiles. Van der Poll et al. [20] have extensively characterized the immunological heterogeneity of sepsis, identifying distinct patient subgroups with different immune profiles. Antcliffe et al. [21] further demonstrated, using transcriptomics, that patients classified into different endotypes (SRS1 and SRS2) exhibit differential responses to hydrocortisone treatment—SRS1 patients showed faster shock reversal, while SRS2 patients derived no similar benefit. These findings underscore the importance of moving beyond static scoring systems toward multidimensional characterization. In this regard, our prior SMART framework [22], based on coagulation–inflammation interactions, supports the value of integrating pathophysiological domains to identify clinically meaningful subgroups and improve risk stratification.

Sepsis is a complex and heterogeneous syndrome with diverse underlying pathophysiological processes, which cannot be fully captured by static, single-time-point assessments. Despite SOFA-2’s effectiveness in reflecting organ dysfunction and its established role in predicting mortality, it remains a scoring system focused solely on organ dysfunction. As such, it inherits key limitations from SOFA, particularly the lack of evaluation of immunological status and insufficient coverage of certain pathophysiological aspects, such as gastrointestinal dysfunction, intestinal barrier injury, and gut microbiota disturbances. As noted by Ranzani et al. [5], SOFA-2 does not capture critical elements of sepsis pathophysiology, including immunosuppression, hyperinflammation, or gastrointestinal dysfunction. Future definitions of sepsis may therefore need to integrate dynamic phenotypes, combining clinical trajectories, biological markers, and electronic health record data, as proposed by Maslove et al. [23]. Within this evolving framework, SOFA-2 could be better understood as one component within a broader, multi-parameter system, rather than as a standalone tool.

This study has several limitations. First, its single-center retrospective design may introduce selection and information biases, limiting generalizability. While the study provides valuable insights, the findings should be interpreted with caution due to the potential biases inherent in a single-center design. As detailed in the discussion, the potential methodological circularity and incorporation bias—stemming from the use of SOFA-based criteria as the reference standard—necessitate viewing these results as an assessment of classification alignment rather than an independent diagnostic validation. Second, the modest sample size and the relatively low number of mortality events restricted statistical power, especially for subgroup analyses and multivariable adjustments. The limited sample size may have prevented us from detecting certain differences, thereby reducing the generalizability of the results. Furthermore, since all scores were calculated using the worst physiological values within the first 24 h, the findings primarily reflect disease severity and prognostic risk, which may not fully represent SOFA-2’s performance for early identification at the immediate point of admission. Third, retrospective studies inherently carry the risk of data incompleteness and potential biases, which may influence the findings. To address these limitations, multicenter prospective studies with larger sample sizes and a focus on initial admission values are needed to validate our findings and further investigate the role of SOFA-2 in sepsis classification and risk stratification.

## 5. Conclusions

This study provides supportive evidence for the clinical utility of the SOFA-2 score in a cohort of patients with suspected infection, showing excellent classification agreement with the current Sepsis-3 standard (SOFA ≥ 2) and superior specificity for mortality prediction compared to SOFA, NEWS2, and SIRS. The strong dose–response relationships between SOFA-2 scores and both in-hospital mortality and ICU stays suggest its potential utility in reflecting disease severity. Furthermore, the recalibrated organ dysfunction scoring—particularly for respiratory and cardiovascular systems—better aligns with contemporary clinical practices. These findings suggest that SOFA-2 is not limited to prognostic assessment of mortality in critically ill ICU populations but may also have potential, like SOFA-1, as a promising candidate scoring system for future sepsis definitions and clinical identification. However, given the single-center retrospective design, multicenter prospective studies are warranted to determine whether SOFA-2 may have a role in future sepsis definitions.

## Figures and Tables

**Table 1:** Baseline characteristics of patients with suspected infection, stratified by sepsis status according to SOFA-1 and SOFA-2 criteria.

Items	SOFA-1		*p* Value	SOFA-2		*p* Value
	Sepsis (*n* = 269)	Non-Sepsis (*n* = 247)		Sepsis (*n* = 249)	Non-Sepsis (*n* = 267)	
Basic characteristics						
Sex, male, *n* (%)	182 (67.7)	130 (52.6)	<0.001 *	172 (69.10)	140 (52.40)	<0.001 *
Age, median (IQR)	71 (60, 79)	65 (43, 77)	<0.001 *	71 (60, 79)	65 (43, 77)	<0.001 *
Mortality	16.4%	2.0%	<0.001 *	17.30%	2.2%	<0.001 *
Clinical biomarker						
WBC, median (IQR)	11.61 (7.8, 16.85)	10.97 (8.3, 14.33)	0.173	11.90 (8.21, 17.10)	10.70 (8.00, 14.11)	0.01 *
Hb, median (IQR)	116 (95.5, 136)	127 (112, 143)	<0.001 *	115 (95.50, 136)	126 (111, 142)	<0.001 *
N%, median (IQR)	89.9 (84.35, 93.1)	84.3 (77.7, 89.4)	<0.001 *	90.00 (84.25, 93.15)	85.20 (78.30, 89.60)	<0.001 *
N#, median (IQR)	10.5 (6.72, 15.25)	9.15 (6.50, 12.27)	0.005 *	10.80 (6.90, 15.70)	8.84 (6.28, 12.16)	<0.001 *
PLT, median (IQR)	160 (126, 225)	198 (165, 242)	<0.001 *	158 (124, 223.5)	198 (162, 242)	<0.001 *
Lym, median (IQR)	0.53 (0.37, 0.85)	1.03 (0.65, 1.58)	<0.001 *	0.54 (0.39, 0.86)	0.99 (0.61, 1.54)	<0.001 *
CRP, median (IQR)	106 (43.99, 154.5)	14.37 (2.80, 38.47)	<0.001 *	111 (52.05, 156.35)	15 (3.0, 47.3)	<0.001 *
PCT, median (IQR)	3.24 (0.83, 18.79)	0.09 (0.02, 0.26)	<0.001 *	3.62 (0.89, 21.60)	0.1 (0.02, 0.30)	<0.001 *
IL-6, median (IQR)	388 (166.91, 1412.5)	74 (22.01, 132.64)	<0.001 *	388 (171.10, 1412.50)	78 (23.30, 146.51)	<0.001 *
OI, median (IQR)	272 (198.5, 351.5)	418 (343, 461)	<0.001 *	265 (193.50, 349.50)	412 (337, 458)	<0.001 *
Lac, median (IQR)	1.90 (1.20, 3.20)	1.40 (1.00, 1.90)	<0.001 *	1.90 (1.20, 3.20)	1.50 (1.00, 1.90)	<0.001 *
ALT, median (IQR)	17 (12, 40.5)	19 (12, 30)	0.373	18 (12, 43)	18 (11, 30)	0.092
AST, median (IQR)	29 (20, 60)	21 (18, 30)	<0.001 *	30 (20, 73)	21 (18, 30)	<0.001 *
TBil, median (IQR)	16.3 (10.10, 26.15)	12.2 (9.10, 16.10)	<0.001 *	16.60 (10.40, 26.50)	12.10 (9.10, 16.20)	<0.001 *
DBil, median (IQR)	7.2 (4.20, 13.65)	3.4 (2.40, 5.00)	<0.001 *	7.60 (4.50, 13.95)	3.40 (2.40, 5.1)	<0.001 *
Alb, median (IQR)	32.4 (26.8, 36.9)	39.8 (35.3, 43.1)	<0.001 *	31.80 (26.50, 36.75)	39.40 (35.30, 43.10)	<0.001 *
Cr, median (IQR)	99 (72, 152.5)	74 (60, 89)	<0.001 *	101 (73.5, 160)	74 (60, 90)	<0.001 *
BUN, median (IQR)	8.7 (6.3, 12.50)	5.7 (4.3, 7.40)	<0.001 *	8.80 (6.35, 12.60)	5.70 (4.30, 7.70)	<0.001 *
PT, median (IQR)	15.20 (14.10, 16.70)	13.40 (12.80, 14.10)	<0.001 *	15.40 (14.20, 16.80)	13.50 (12.80, 14.20)	<0.001 *
APTT, median (IQR)	40.70 (35.45, 47.15)	33.90 (31.00, 37.10)	<0.001 *	41.70 (36.00, 48.10)	34.10 (31.00, 37.10)	<0.001 *
INR, median (IQR)	1.21 (1.11, 1.36)	1.03 (0.97, 1.10)	<0.001 *	1.24 (1.11, 1.38)	1.03 (0.97, 1.10)	<0.001 *
Fig, median (IQR)	4.79 (3.46, 6.32)	3.58 (2.82, 4.58)	<0.001 *	4.81 (3.48,6.37)	3.60 (2.87, 4.59)	<0.001 *
D-dimer, median (IQR)	3.49 (2.24, 5.47)	1.16 (0.60, 1.92)	<0.001 *	3.50 (2.26, 5.52)	1.18 (0.65, 2.12)	<0.001 *
Site of infection						
Lung, *n* (%)	103 (38.30%)	79 (32.00%)	<0.001 *	95 (38.20%)	87 (32.60%)	<0.001 *
Abdominal cavity, *n* (%)	117 (43.50%)	90 (36.40%)		107 (43.00%)	100 (37.50%)	
Urinary tract, *n* (%)	18 (6.70%)	39 (15.80%)		17 (6.80%)	40 (15.00%)	
Skin, *n* (%)	16 (5.90%)	21 (8.50%)		15 (6.00%)	22 (8.20%)	
Others, *n* (%)	15 (5.60%)	18 (7.30%)		15 (6.00%)	18 (6.70%)	

SOFA-1, Sequential Organ Failure Assessment-1; SOFA-2, Sequential Organ Failure Assessment-2; WBC, White Blood Cell; Hb, Hemoglobin; N%, Neutrophil Ratio; N#, Neutrophil count; PLT, Platelet; Lym, Lymphocyte; CRP, C-Reactive Protein; PCT, Procalcitonin; IL-6, Interleukin-6; OI, Oxygenation Index; Lac, Lactate; ALT, Alanine Transaminase; AST, Aspartate Transaminase; TBil, Total Bilirubin; DBil, Direct Bilirubin; Alb, Albumin; Cr, Creatinine; BUN, Blood Urea Nitrogen; PT, Prothrombin time; APTT, Activated partial thromboplastin time; INR, International Normalized Ratio; Fig, Fibrinogen. * indicates a significant value, *p* < 0.05.

**Table 2:** Distribution of organ dysfunction scores in SOFA-1 and SOFA-2 criteria.

Organ System	SOFA-1 Score					SOFA-2 Score				
	0	1	2	3	4	0	1	2	3	4
Brain	400	65	32	7	12	400	65	32	7	12
Respiratory	221	153	81	49	12	336	93	52	30	5
Cardiovascular	351	12	17	52	84	352	13	86	27	38
Liver	397	81	31	7	0	400	91	19	6	0
Kidney	382	88	31	14	1	395	69	37	11	4
Hemostasis	363	128	19	5	1	365	124	17	6	4

SOFA-1, Sequential Organ Failure Assessment-1; SOFA-2, Sequential Organ Failure Assessment-2.

## Data Availability

The datasets used are available from the corresponding author upon reasonable request.

## Supplementary Materials

The following supporting information can be downloaded at: https://www.mdpi.com/article/10.3390/diagnostics16111579/s1, Figure S1. Sankey diagram illustrating patient reclassification among SOFA, SOFA-2, and NEWS2 criteria. Figure S2. In-hospital mortality rates according to the adjusted SOFA-2 score in patients with suspected infection. Figure S3. Correlation between SOFA-2 score and hospital stay (scatter plot with linear fit). Table S1. Characteristics of patients with suspected infection stratified by SIRS criteria. Table S2. 2 × 2 contingency table comparing SOFA-2 and Sepsis-3 criteria for sepsis diagnosis.

## References

[1] Seymour C.W., Liu V.X., Iwashyna T.J., Brunkhorst F.M., Rea T.D., Scherag A., Rubenfeld G., Kahn J.M., Shankar-Hari M., Singer M. (2016). Assessment of Clinical Criteria for Sepsis: For the Third International Consensus Definitions for Sepsis and Septic Shock (Sepsis-3). JAMA.

[2] Prescott H.C., Antonelli M., Alhazzani W., Møller M.H., Alshamsi F., Azevedo L.C.P., Belley-Cote E., De Waele J., Derde L., Dionne J.C. (2026). Surviving Sepsis Campaign: International Guidelines for Management of Sepsis and Septic Shock 2026. Crit. Care Med..

[3] Bone R.C., Balk R.A., Cerra F.B., Dellinger R.P., Fein A.M., Knaus W.A., Schein R.M.H., Sibbald W.J. (1992). Definitions for Sepsis and Organ Failure and Guidelines for the Use of Innovative Therapies in Sepsis. Chest.

[4] Levy M.M., Fink M.P., Marshall J.C., Abraham E., Angus D., Cook D., Cohen J., Opal S.M., Vincent J.L., Ramsay G. (2003). 2001 SCCM/ESICM/ACCP/ATS/SIS International Sepsis Definitions Conference. Crit. Care Med..

[5] Ranzani O.T., Singer M., Salluh J.I.F., Shankar-Hari M., Pilcher D., Berger-Estilita J., Coopersmith C.M., Juffermans N.P., Laffey J., Reinikainen M. (2025). Development and Validation of the Sequential Organ Failure Assessment (SOFA)-2 Score. JAMA.

[6] Pei F., Gu B., Liu Z., Li G., Nie Y., Chen M., Liu Y., Guan X., Chen Q., Wu J. (2026). Validation of SOFA-2 score in sepsis and exploration of its extension with additional immune markers. J. Intensive Med..

[7] Evans L., Rhodes A., Alhazzani W., Antonelli M., Coopersmith C.M., French C., Machado F.R., McIntyre L., Ostermann M., Prescott H.C. (2021). Surviving sepsis campaign: International guidelines for management of sepsis and septic shock 2021. Intensive Care Med..

[8] Williams B. (2022). The National Early Warning Score: From concept to NHS implementation. Clin. Med..

[9] Vincent J.L., Moreno R., Takala J., Willatts S., De Mendonça A., Bruining H., Reinhart C.K., Suter P.M., Thijs L.G. (1996). The SOFA (Sepsis-related Organ Failure Assessment) score to describe organ dysfunction/failure. On behalf of the Working Group on Sepsis-Related Problems of the European Society of Intensive Care Medicine. Intensive Care Med..

[10] Kumar A., Roberts D., Wood K.E., Light B., Parrillo J.E., Sharma S., Suppes R., Feinstein D., Zanotti S., Taiberg L. (2006). Duration of hypotension before initiation of effective antimicrobial therapy is the critical determinant of survival in human septic shock. Crit. Care Med..

[11] Churpek M.M., Zadravecz F.J., Winslow C., Howell M.D., Edelson D.P. (2015). Incidence and Prognostic Value of the Systemic Inflammatory Response Syndrome and Organ Dysfunctions in Ward Patients. Am. J. Respir. Crit. Care Med..

[12] Singer M., Deutschman C.S., Seymour C.W., Shankar-Hari M., Annane D., Bauer M., Bellomo R., Bernard G.R., Chiche J.D., Coopersmith C.M. (2016). The Third International Consensus Definitions for Sepsis and Septic Shock (Sepsis-3). JAMA.

[13] Angus D.C., van der Poll T. (2013). Severe sepsis and septic shock. N. Engl. J. Med..

[14] Price C., Prytherch D., Kostakis I., Briggs J. (2023). Evaluating the performance of the National Early Warning Score in different diagnostic groups. Resuscitation.

[15] Marosi V., Gadda G., Timoftica C., Alberti A., Destrebecq A. (2023). National Early Warning Score 2. to Identify Sepsis in the Emergency Department. A Review of the Literature. J. Med. Clin. Nurs. Stud..

[16] Wacker C., Prkno A., Brunkhorst F.M., Schlattmann P. (2013). Procalcitonin as a diagnostic marker for sepsis: A systematic review and meta-analysis. Lancet Infect. Dis..

[17] Bakker J., Nijsten M.W., Jansen T.C. (2013). Clinical use of lactate monitoring in critically ill patients. Ann. Intensive Care.

[18] Nguyen H.B., Rivers E.P., Knoblich B.P., Jacobsen G., Muzzin A., Ressler J.A., Tomlanovich M.C. (2004). Early lactate clearance is associated with improved outcome in severe sepsis and septic shock. Crit. Care Med..

[19] Liu B., Du H., Zhang J., Jiang J., Zhang X., He F., Niu B. (2022). Developing a new sepsis screening tool based on lymphocyte count, international normalized ratio and procalcitonin (LIP score). Sci. Rep..

[20] van der Poll T., Shankar-Hari M., Wiersinga W.J. (2021). The immunology of sepsis. Immunity.

[21] Antcliffe D.B., Burnham K.L., Al-Beidh F., Santhakumaran S., Brett S.J., Hinds C.J., Ashby D., Knight J.C., Gordon A.C. (2019). Transcriptomic Signatures in Sepsis and a Differential Response to Steroids. From the VANISH Randomized Trial. Am. J. Respir. Crit. Care Med..

[22] Zhu L., Chen Z., Zhang H., Chen H., Liu L., Yu W., Wu K., Chen Y., Tao X., Yu Z. (2025). Explainable AI unravels sepsis heterogeneity via coagulation-inflammation profiles for prognosis and stratification. Nat. Commun..

[23] Maslove D.M., Tang B., Shankar-Hari M., Lawler P.R., Angus D.C., Baillie J.K., Baron R.M., Bauer M., Buchman T.G., Calfee C.S. (2022). Redefining critical illness. Nat. Med..

