# Supplementary material for: A Comparative Evaluation of SIRS, NEWS2, SOFA, and the Novel SOFA-2 Score for Sepsis Classification Agreement and Outcome Prediction
Source: Diagnostics (Basel). 2026 May 23;16(11):1579. doi: 10.3390/diagnostics16111579 (PMC13257333; doi:10.3390/diagnostics16111579)

Supplementary Materials

Table S1. Characteristics of patients with suspected infection stratified by SIRS criteria. 2
Table S2. 2×2 contingency table comparing SOFA-2 and Sepsis-3 criteria for sepsis diagnosis. 4

Figure S1. Sankey diagram illustrating patient reclassification among SOFA, SOFA-2, and NEWS2 criteria. 5

Figure S2. In-hospital mortality rates according to the adjusted SOFA-2 score in patients with suspected infection. 6

Figure S3. Correlation between SOFA-2 score and hospital stay........ 7

Table S1. Characteristics of patients with suspected infection stratified by SIRS criteria.

**Items**	**SIRS**		***P* value**
	**Sepsis(*n*=319)**	**Non-sepsis(*n*=197)**	
Basic characteristics			
Sex, male, n (%)	202(63.3)	110(55.8)	0.096
Age, median (IQR)	71(58,79)	64(46,76)	0.002*
Mortality	14.1%	2%	＜0.001*
Clinical biomarker			
WBC, median (IQR)	12.5(8.29, 16.7)	10(7.91, 13.01)	＜0.001*
Hb,median (IQR)	118(101, 136)	125(111, 143)	＜0.001*
N%, median (IQR)	88.8(83.7, 92.5)	84.2(76.7, 89.4)	＜0.001*
N#, median (IQR)	10.9(7, 14.8)	8.4(6.01, 11.16)	＜0.001*
PLT, median (IQR)	177(132, 236)	192(156, 235)	0.032
Lym, median (IQR)	0.63(0.4, 1)	1.01(0.59, 1.57)	＜0.001*
CRP, median (IQR)	82.8(21.7, 141)	16.82(2.38, 57)	＜0.001*
PCT, median (IQR)	1.5(0.26,12.2)	0.09(0.02,0.34)	＜0.001*
IL-6, median (IQR)	285(101, 1162)	76.21(19.81, 146.08)	＜0.001*
OI, median (IQR)	297(215.38, 381)	413(340.69, 457)	＜0.001*
Lac, median (IQR)	1.73(1.2, 3.1)	1.4(1.0, 1.8)	＜0.001*
ALT, median (IQR)	17(12, 34)	20(11, 34)	0.929
AST, median (IQR)	26(19, 51)	21(18, 31.5)	＜0.001*
TBil, median (IQR)	14.9(9.8, 22.9)	12.5(9.4, 17.3)	0.01*
DBil, median (IQR)	6.1(3.5, 11.3)	3.6(2.6, 5.4)	＜0.001*
Alb, median (IQR)	33.9(28.2, 39.1)	39.3(35, 43)	＜0.001*
Cr, median (IQR)	88(70, 141)	76(61.5, 93)	＜0.001*
BUN, median (IQR)	8.2(5.4, 11.7)	5.9(4.55, 7.8)	＜0.001*
PT, median (IQR)	14.8(13.7, 16.4)	13.5(12.8, 14.4)	＜0.001*
APTT, median (IQR)	38.8(34.1, 45.8)	34.7(31.4, 37.9)	＜0.001*
INR, median (IQR)	1.16(1.06, 1.33)	1.04(0.97, 1.12)	＜0.001*
Fig, median (IQR)	4.6(3.39, 5.97)	3.52(2.82, 4.45)	＜0.001*
D-dimer, median (IQR)	3.12(1.24, 5.07)	1.13(0.69, 2.4)	＜0.001*

Items	SIRS		*P* value
	**Sepsis(*n*=319)**	**Non-sepsis(*n*=197)**	
Site of infection			
Lung, n (%)	136(42.6%)	46(23.4%)	
Abdominal cavity, n (%)	124(38.9%)	83(42.1%)	
Urinary tract, n (%)	22(6.9%)	35(17.8%)	＜0.001*
Skin, n (%)	18(5.6%)	19(9.6%)	
Others, n (%)	19(6%)	14(7.1%)	

SIRS, Systemic Inflammatory Response Syndrome; WBC, White Blood Cell; Hb, Hemoglobin; N%, Neutrophil Ratio; N#, Neutrophil count; PLT, Platelet; Lym, Lymphocyte; CRP, C-Reactive Protein; PCT, Procalcitonin; IL-6, Interleukin-6; OI, Oxygenation Index; Lac, Lactate; ALT, Alanine Transaminase; AST, Aspartate Transaminase; TBil, Total Bilirubin; DBil, Direct Bilirubin; Alb, Albumin; Cr, Creatinine; BUN, Blood Urea Nitrogen; PT, Prothrombin time; APTT, Activated partial thromboplastin time; INR, International Normalized Ratio; Fig, Fibrinogen.* indicates a significant value, $P<0.05$.

Table S2. 2×2 contingency table comparing SOFA-2 and Sepsis-3 criteria for sepsis diagnosis.

		Sepsis-3 Criteria		Kappa	*p* value
		**SOFA-1(≥2)**	**SOFA-1(＜2)**		
**SOFA-2 Criteria**	**SOFA-2(≥2)**	249	0	0.923	<0.001*
	**SOFA-2(< 2)**	20	247		

SOFA-1, Sequential Organ Failure Assessment-1; SOFA-2, Sequential Organ Failure Assessment-2; SOFA-1 (≥2) was used as the reference standard. The Cohen's κ was calculated to assess agreement between the two criteria. * indicates a significant value, $P < 0.05$.

Figure S1. Sankey diagram illustrating patient reclassification among SOFA, SOFA-2, and NEWS2 criteria.

The diagram visualizes the flow of patients classified as septic by each scoring system at their respective diagnostic thresholds (SOFA ≥2, SOFA-2 ≥2, NEWS2 ≥5), demonstrating the degree of overlap and divergence in patient assignment across the three tools.

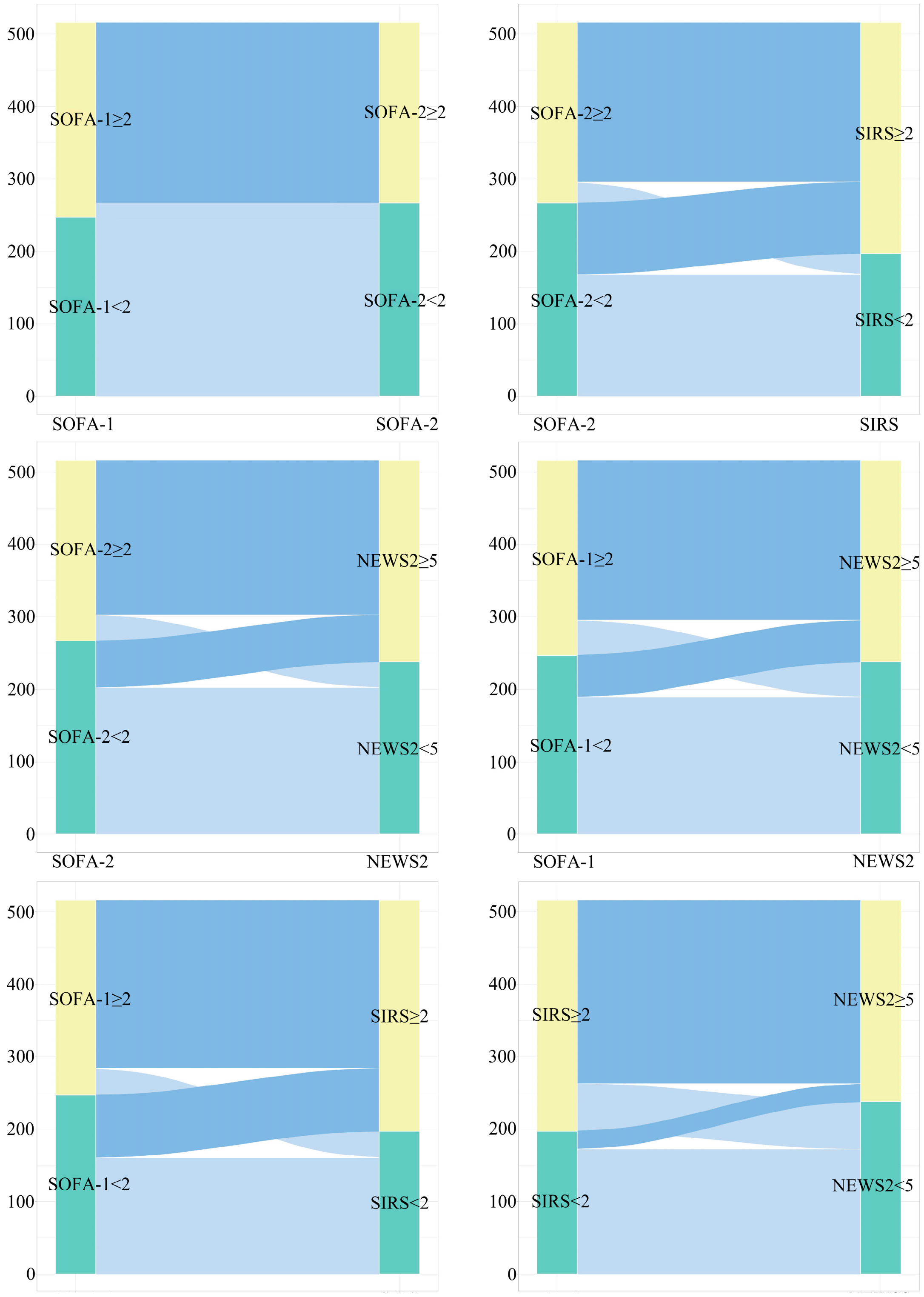

Figure S2. In-hospital mortality rates according to the adjusted SOFA-2 score in patients with suspected infection.

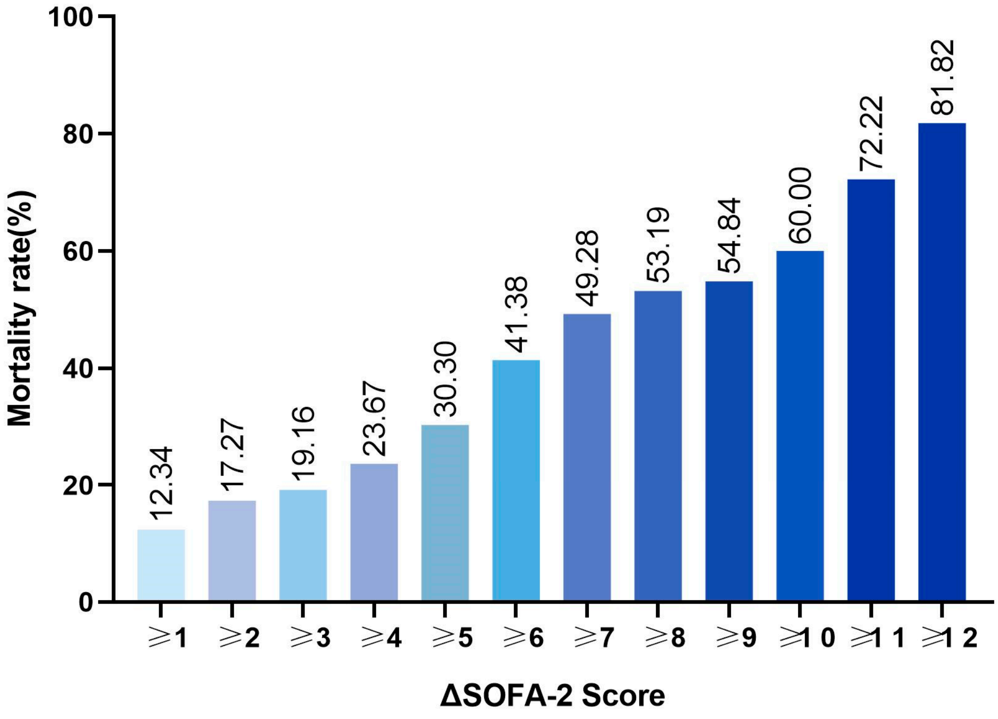

Figure S3. Correlation between SOFA-2 score and hospital stay (scatter plot with linear fit).

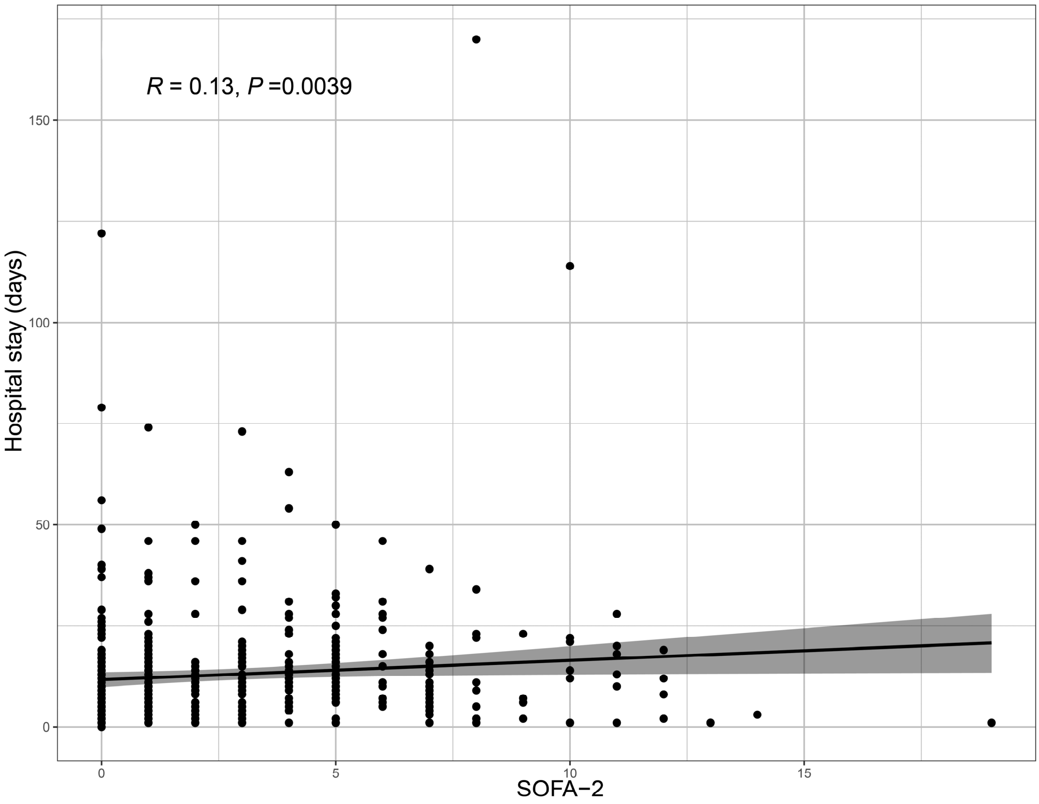

Supplement: Supplementary file 1 [file diagnostics-16-01579-s001.zip › diagnostics-4245981-supplementary.pdf]
